# Local prothoracic auditory neurons in Ensifera

**DOI:** 10.3389/fnins.2022.1087050

**Published:** 2022-12-21

**Authors:** Ali Cillov, Andreas Stumpner

**Affiliations:** Department of Cellular Neurobiology, Johann-Friedrich-Blumenbach-Institute of Zoology & Anthropology, University of Göttingen, Göttingen, Germany

**Keywords:** orthoptera, bush cricket, neuronal processing, acoustic communication, local neurons

## Abstract

A new method for individually staining insect neurons with metal ions was described in the late 60s, closely followed by the introduction of the first bright fluorescent dye, Lucifer Yellow, for the same purpose. These milestones enabled an unprecedented level of detail regarding the neuronal basis of sensory processes such as hearing. Due to their conspicuous auditory behavior, orthopterans rapidly established themselves as a popular model for studies on hearing (first identified auditory neuron: 1974; first local auditory interneuron: 1977). Although crickets (Ensifera, Gryllidae) surpassed grasshoppers (Caelifera) as the main model taxon, surprisingly few neuronal elements have been described in crickets. More auditory neurons are described for bush crickets (Ensifera, Tettigoniidae), but due to their great biodiversity, the described auditory neurons in bush crickets are scattered over distantly related groups, hence being confounded by potential differences in the neuronal pathways themselves. Our review will outline all local auditory elements described in ensiferans so far. We will focus on one bush cricket species, *Ancistrura nigrovittata* (Phaneropterinae), which has the so-far highest diversity of identified auditory interneurons within Ensifera. We will present one novel and three previously described local prothoracic auditory neuron classes, comparing their morphology and aspects of sensory processing. Finally, we will hypothesize about their functions and evolutionary connections between ensiferan insects.

## Introduction

Orthopterans (crickets, bush crickets/katydids, grasshoppers, and allies) exhibit an enormous variation of lifestyles. They can live in habitats as different as burrows and caves or the forest canopy, can be nocturnal or diurnal, flying or flightless. Yet, the majority uses acoustic signals for intraspecific communication and/or predator detection (e.g., Desutter-Grandcolas, 2003; Song et al., 2020). Their conspicuous behavior has intrigued researchers early on and some of the pioneering studies on insect hearing were done with orthopterans (Regen, 1913, 1914; Autrum, 1940; also see Gogala, 2014 for a summary of the early research history). After Roeder’s studies on hearing in moths (e.g., Roeder, 1966) demonstrated the potential for inferring behavior from neuronal activity, researchers started to study the neuronal basis of orthopteran hearing as well. Repeated recordings of the same physiological responses in different individuals brought about the need to identify these units morphologically. Staining cells with cobalt salts during extracellular recordings was the preferred technique (e.g., Rehbein et al., 1974). However, this method does not allow unambiguous matching of the recorded and the stained cell and was replaced by staining with Lucifer Yellow. Developed by Stewart (1978), Lucifer Yellow was the first commercially successful fluorescent dye. It was easy to apply by hyperpolarizing current and about 100 times more effective than its predecessor (Procion Yellow; Stretton and Kravitz, 1968), enabling very detailed morphological observations. The first publication showing auditory neurons stained with Lucifer Yellow came from Wohlers and Huber (1982) on six cricket interneurons, followed by studies on the neuronal basis of insect acoustic communication (for an outline, see Hoy et al., 1998; Hedwig, 2014), with a special focus on ensiferans (bush crickets: Bailey and Rentz, 1990; crickets: Huber et al., 1989). The increasing availability of confocal microscopes in the 1990s, coupled with a plethora of new fluorescent dyes, made multiple cell stains possible (e.g., Imaizumi and Pollack, 1999; Molina and Stumpner, 2005; Lefebvre et al., 2018). Thus, the “identified neuron concept” (Hoyle, 1983), characterizing cells so that they are recognizable by their anatomical and physiological characteristics in different individuals, became the dominant approach in insect neuroscience.

Ensiferan ears are located in the forelegs. Each foreleg tibia bears two tympanic membranes (an anterior and a posterior one) that are either open to the surrounding environment or covered with cuticular flaps. The tympana are mostly similar in size in bush crickets while one tympanum is often reduced in size and non-functional in many cricket species (Larsen et al., 1989; Mhatre et al., 2009). The tympana are coupled to the underlying branches of the acoustic trachea, which runs through the leg into the thorax, where it terminates at the (often greatly enlarged) acoustic spiracle in the mesothorax, thereby constituting another input for sound waves into the acoustic system (Hill and Boyan, 1976; Larsen and Michelsen, 1978). The auditory tracheae on the left and right are always connected in the thorax in crickets (Schmidt and Römer, 2016) and may be functionally coupled in bush crickets (Bailey, 1990). The sensory organ (called crista acustica in bush crickets) contains tonotopically organized scolopidia with sensory axons projecting exclusively into the prothoracic “auditory neuropile” (= anterior ring tract, Lakes and Schikorski, 1990). There, the sensory terminals connect to local, descending, ascending and T-fibers. Much of the final sound processing (e.g., song recognition, predator detection) likely happens in the brain (Huber and Thorson, 1985; Stumpner and Nowotny, 2014; Pollack and Hedwig, 2017).

Over decades, certain topics and phenomena (e.g., frequency and pattern coding, directionality, neuronal activity during behavior) established themselves as focal points for research on the ensiferan auditory system. One example, on the peripheral level, is the biophysical dynamics in the hearing organ and the tonotopy of sensory neurons (Oldfield, 1988; Michelsen et al., 1994; Imaizumi and Pollack, 1999; Schul and Patterson, 2003; Montealegre-Z et al., 2012; Vavakou et al., 2021). Another example and the biggest focus in terms of research interest is interneurons, especially the omega neuron 1 (in crickets *Acheta*: Atkins et al., 1984; Stumpner et al., 1995; *Gryllus*: Popov et al., 1978; Wohlers and Huber, 1982; Schildberger and Hörner, 1988; Hardt and Watson, 1994; *Teleogryllus*: Hennig, 1988; Faulkes and Pollack, 2001; in mole crickets *Scapteriscus*: Mason et al., 1998; in grigs *Cyphoderris*: Mason and Schildberger, 1993; in bush crickets *Ancistrura*: Molina and Stumpner, 2005; Stumpner and Molina, 2006; *Mecopoda*: Römer et al., 2002; Kostarakos and Römer, 2015; *Mygalopsis*: Römer and Bailey, 1986; Römer, 1987; *Neoconocephalus*: Triblehorn and Schul, 2009; Prešern et al., 2015; *Tettigonia*: Schul, 1997; Römer and Krusch, 2000). Together with the interneurons ascending to the brain, the song recognition network in the cricket brain has also attracted significant attention. Early work by Schildberger (1984) became the textbook example for a neuronal band-pass filter for temporal pattern extraction, but it was recently replaced by another concept and set of brain neurons as the most likely candidate for song recognition (Schöneich et al., 2015). Though technically demanding, even integrative aspects of the ensiferan nervous system have been investigated, such as initiating behavior by activation of single neurons (Nolen and Hoy, 1984) and corollary discharge dynamics during singing (Poulet and Hedwig, 2002, 2006).

While studies on ensiferan hearing became increasingly complex – from counting spikes to extracting information rates—there are still gaps in our knowledge regarding some basic points (e.g., transmitters of the described neurons, sources of inhibition). Although described first, the neurotransmitter of the omega neuron is still unclear. Moreover, very little is known as to how local circuits in thoracic ganglia shape the information relayed to the brain. Perhaps more fundamentally, properties of known neurons suggest that not all auditory units in these ganglia have been discovered yet (e.g., Stumpner, 1999; Faulkes and Pollack, 2001). Below, we will present a complete overview of the local prothoracic auditory neurons described in various ensiferan species and introduce two new elements in bush crickets.

## Local prothoracic neurons in Ensifera—An overview

**FIGURE 1:**
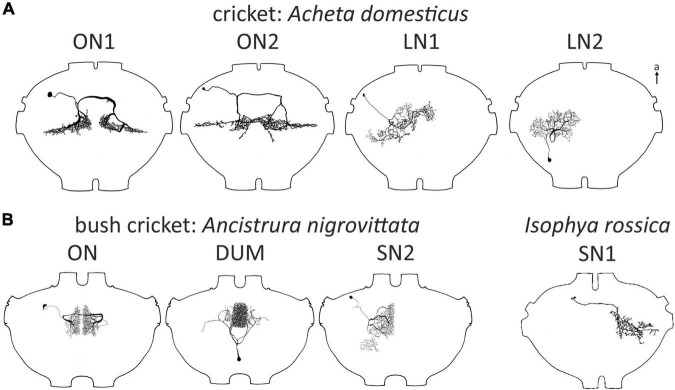
Local auditory neurons in crickets **(A)** and bushrickets **(B)**. All types also in the bush cricket *A. nigrovittata* except for SN1 described in *Isophya rossica*, which has not been described in any other Ensiferan. All ganglia of similar size but not exactly drawn to scale for better comparability. a, anterior; ax, axon. LN1, LN2 redrawn with permission after Stiedl et al. (1997), SN1 redrawn after Zhantiev and Korsunovskaya (1990b).

In Ensifera, sensory cells of the ear in the foreleg tibia project exclusively into the prothoracic ganglion (TG1) (Rehbein, 1973). Thus, auditory information is first processed in TG1 and the local circuitry has to be considered when studying the neuronal basis of auditory behavior. Consequently, TG1 houses the highest diversity of identified auditory neurons in the central nervous system of ensiferans (see Table 1). The first such interneuron was the omega neuron 1 (ON1, see Figure 1A), initially named “large segmental auditory neuron”. ON1 was first described in *Gryllus bimaculatus* by Andjan in 1976, and published in Popov et al. (1978). The discovery of the “homologous” neuron in *Teleogryllus oceanicus* happened simultaneously (Casaday and Hoy, 1977). A possible role of ON1 in directional hearing was suggested and finally demonstrated in 1985 (Selverston et al., 1985; Wiese and Eilts, 1985). The first report describing ON1 in a bush cricket (Figure 1B) came in 1983 (*Tettigonia cantans*, Zhantiev and Korsunovskaya, 1983) and its existence was demonstrated in further taxa in the following decade (grigs: Mason and Schildberger, 1993; mole crickets: Zhantiev and Korsunovskaya, 1990a, Figure 2B). ON1 described in different species have never been directly shown to be homologous, which would require its demonstration in a common ancestor or proof of a common developmental origin. Yet, their presence in a great number of orthopterans and the undeniable similarities in physiology and morphology make a compelling case for homology.

**TABLE 1:** Types of local neurons and names as published for different taxa.

Neuron type	Name	Taxon	First reference
Omega	Omega neuron 1, ON1	Crickets	Wohlers and Huber, 1978
	Omega-neuron, ON	Bush crickets	Zhantiev and Korsunovskaya, 1983
	ON1	Mole crickets (*Gryllotalpa*)	Zhantiev and Korsunovskaya, 1990a
	LON (low-frequency tuned ON)	Mole crickets (*Scapteriscus*)	Mason et al., 1998
	HON (high-frequency tuned ON)	Mole crickets (*Scapteriscus*)	Mason et al., 1998
	Omega neuron 2, ON2	Crickets	Wohlers and Huber, 1982
Dorsal unpaired median	DUM	Bush crickets (*Ancistrura*)	Lefebvre et al., 2018
Segmental	LN1	Crickets (*Acheta*)	Stiedl et al., 1997
	LN2	Crickets (*Acheta*)	Stiedl et al., 1997
	SN1	Bush crickets (*Isophya*)	Zhantiev and Korsunovskaya, 1990b
	SN2	Bush crickets	Stumpner, 1995
Local descending	LDN	Bush crickets (*Ancistrura*)	This publication

If a neuron was only encountered in one species or genus, the genus name is given as well. The reference only cites the publication, in which this name was given for the first time.

**FIGURE 2:**
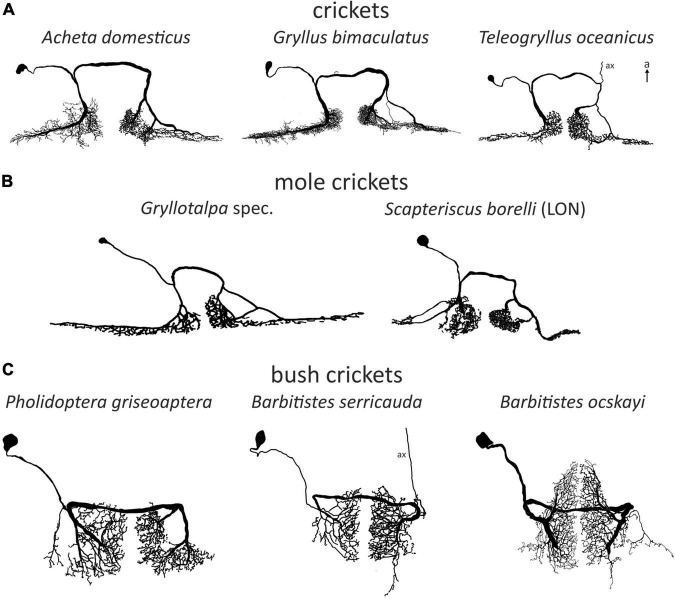
Examples of omega (1) neurons in Ensifera. Soma-ipsilateral branches have a fine dendritic structure, soma-contralateral branches have a more beaded appearance typical for axonic arborizations. **(A)** Field crickets. *T. oceanicus* modified with permission after Atkins and Pollack (1986). **(B)** Mole crickets. In *Scapteriscus* there are two ON1-like morphologies, one is interpreted as more ON1-like (“high-frequency-tuned omega neuron”). *Gryllotalpa* modified after Zhantiev and Korsunovskaya (1990a); *Scapteriscus* modified with permission after Mason et al. (1998). **(C)** Bush crickets. Neurons not drawn to scale for better comparability. a, anterior; ax, axon.

Another interneuron roughly similar to, but consistently different from ON1 in *Gryllus campestris* was named ON2 (Wohlers and Huber, 1982; Figure 1A). Yet, the physiological responses of ON2 reported in different studies proved to be controversial (see below for a detailed discussion). Based on a double staining, Mason and Schildberger (1993) proposed the presence of ON2 in the grig *Cyphoderris monstrosa*, but this finding leaves room for interpretation. Unlike ON1, ON2 was never described for any bush cricket species.

Gras et al. (1990) presented DUM neurons (dorsal unpaired median soma; Hoyle et al., 1974) in *G. bimaculatus* that responded to sound, but these neurons had high response thresholds and multimodal input. Morphologically, they all project into peripheral nerves or other segments. In contrast, a distinctly auditory population of DUM neurons occurs in the bush cricket *Ancistrura nigrovittata* (Lefebvre et al., 2018; Figure 1B). These were proposed to play a role in frequency processing through frequency-specific inhibition. Although frequency-specific inhibition is present in crickets as well, a similar neuron population has not been reported in any cricket species.

Two local neurons, named segmental neuron 1 and 2 (SN; Figure 1B) were described in bush crickets (SN2: *A. nigrovittata*: Stumpner, 1995; SN1: *Isophya rossica*, Zhantiev and Korsunovskaya, 1990b; both without very detailed characterization). SN1 has not been reported in any other ensiferan species, but a neuron described by Stiedl et al. (1997) in *Acheta domesticus* is broadly similar to SN2 (LN1, Figure 1A). Another neuron identified by Stiedl et al. (1997) has also never been reported in any other study in orthopterans (LN2. Figure 1A). This was also the last report of a new local auditory neuron in crickets to date.

The data on local auditory neurons come from different species, spread over several “subfamilies”. Although research on hearing in ensiferans has a history spanning multiple decades, no single species became established as the preferred model. Unfortunately, there are no genetic tools available for any ensiferan species as for *Caenorhabditis elegans*, *Drosophila melanogaster*, or *Tribolium castaneum*. Consequently, insect hearing research shifted in focus from orthopterans to *D. melanogaster* (e.g., Caldwell and Eberl, 2002; Albert and Göpfert, 2015; Clemens et al., 2015). However, similar toolkits as for *Drosophila* are in development for *G. bimaculatus* (Kulkarni and Extavour, 2019). Furthermore, the proliferation of tools such as CRISPR-Cas (Pickar-Oliver and Gersbach, 2019), which can be applied to non-model insects, enables novel approaches to existing questions. These methodological developments may bring new momentum into orthopteran auditory research, furthering our understanding as to how these “simple” insects perceive the sensory world around them and what evolutionary mechanisms underlie this process.

In the following, we will review existing data on all three identified local auditory neurons (i.e., any neuron without branches projecting into other ganglia or into the periphery) in Ensifera, focusing on species differences and potential functions. Additionally, we will present a new local neuron. We hope to convince the reader that even after half a century of research, we are far from understanding the full scope of neuronal processes, even outside the brain and how these drive acoustic perception and communication.

## Omega neurons

Omega neurons, which occur as mirror images on both sides of TG1, are named after their outward similarity to the Greek capital letter Ω. Though this is informative about the morphology, the initial name used by (Popov et al., 1978), large segmental auditory neuron, is a better indicator as to why ON1 is the most intensively studied ensiferan neuron: it has unusually large main branches on both sides of the prothoracic ganglion and a thick crossing segment close to the tissue surface, which makes recordings technically simple. Recordings in soma-ipsilateral branches show strong graded potentials with action potentials, recordings on the soma-contralateral side show mainly action potentials (Wohlers and Huber, 1978), but may show IPSPs as well (Schul, 1997). Early studies found morphologically very similar neurons in different cricket species (*Acheta*: Atkins et al., 1984; *Gryllus*: Popov et al., 1978; *Teleogryllus*: Casaday and Hoy, 1977; see Figure 2A). In bush crickets, all studied species had a similar neuron as well, albeit with ca. 90° rotated arborizations (Zhantiev and Korsunovskaya, 1983; Boyan, 1984; Römer, 1985; compare Figures 2A, C). However, different bush cricket subfamilies vary slightly in the morphology of their ON1. Whereas Tettigoniinae (e.g., *Tettigonia*, *Pholidoptera*, *Metrioptera*) have a more rectangular dendritic tree and a crossing segment lying more anteriorly than the branching area, Phaneropterinae (e.g., *Ancistrura*, *Barbitistes*, *Leptophyes*) and Mecopodinae (*Mecopoda*) have a triangular arborization and the crossing segment appears to be “within” the dendrites (while actually being more ventral, Figure 2C). A neuron similar to ON1 was later also found in further ensiferan taxa, such as mole crickets (*Gryllotalpa*: Zhantiev and Korsunovskaya, 1990a; *Scapteriscus*: Mason et al., 1998) and grigs (*Cyphoderris*: Mason and Schildberger, 1993). The close similarities between omega neurons in various ensiferan groups are surprising, especially considering their significant evolutionary separation (mole crickets vs. true crickets 180–230 mya, bush crickets vs. true crickets 270–300 mya, Song et al., 2015, 2020).

Flying female crickets show two highly directional behaviors: positive phonotaxis toward a singing male and negative phonotaxis away from high-frequency bat echolocation calls (e.g., Pollack et al., 1984; Wyttenbach et al., 1996). The potential role of ON1 in sharpening directional decisions in behavior was alluded to in early studies. This was directly demonstrated for positive phonotaxis by suppressing its activity, though the effects were not fully congruent: besides the high interindividual variability, the effect was also dependent on the stimulus parameters (*Acheta*: Atkins et al., 1984; *Gryllus*: Schildberger and Hörner, 1988). Moreover, inactivating ON1 on both hemiganglia had no effect on positive phonotaxis, though the used measurement methods were not very sensitive in general (Atkins et al., 1984). The neuronal mechanisms underlying ON1’s influence on directional behavior have also been elucidated. Photoinactivation and cell killing experiments show that the mirror image ON1 have strong contralateral mutual inhibition (Selverston et al., 1985; Wiese and Eilts, 1985). In *Teleogryllus*, ascending neuron 2 (AN2, also called Interneuron-1) plays a central role in negative phonotaxis in flight, and is both necessary and sufficient for this behavior (Nolen and Hoy, 1984). Similar to ON1, AN2 is also directionally inhibited (Moiseff and Hoy, 1983; Faulkes and Pollack, 2000). Although Harrison et al. (1988) did not find any connection between ON1 and AN2, Faulkes and Pollack (2000) demonstrated the loss of directional inhibition in AN2 in the same species (*T. oceanicus*) upon inactivation of ON1. Selverston et al. (1985) further demonstrated that AN2 is inhibited by the ON1 that receives excitation from the opposite ear. This inhibition can also affect positive phonotaxis (Schildberger and Hörner, 1988). Recordings of ON1 in bush crickets do not differ significantly from those in crickets and contralateral inhibition is also present as a prominent feature (Römer et al., 1988; Schul, 1997; Römer and Krusch, 2000). However, a combination of photoinactivation, pharmacological blocking, and mechanical ear destruction experiments indicate that there is contralateral inhibition in ON1 in addition to that of the mirror image in *A. nigrovittata* (Molina and Stumpner, 2005).

Electron microscopy studies in *Gryllus* have shown that ON1 receives monosynaptic input from auditory sensory cells (Watson and Hardt, 1996; Hirtz and Wiese, 1997). Data from *Teleogryllus*, however, strongly suggest that only input from high-frequency (HF) receptors is direct, whereas input from low-frequency (LF) receptors is polysynaptic (Faulkes and Pollack, 2001). This leads to a distinctly longer latency at LF, which may be relevant for temporal processing (see below). Furthermore, the distribution of synapses of ON1 is rather complex. Surprisingly, there are significant proportions of both input and output synapses on both sides of the ganglion, which are connected with neurons other than the auditory receptors and the mirror image ON1 (Watson and Hardt, 1996). Many of the inputs into ON1 are immunoreactive for γ-aminobutyric acid (GABA) (e.g., potentially from the observed vibratory inhibition, see Wiese, 1981). Yet, ON1 itself does not use GABA as neurotransmitter, as shown by several studies in both crickets and bush crickets (e.g., Watson and Hardt, 1996; Stumpner et al., 2020). While there is strong functional evidence for histamine as the neurotransmitter of cricket ON1 (Skiebe et al., 1990), immunohistochemical studies failed to confirm this finding (Hörner, 1999). Similar approaches have revealed a morphologically similar, serotonergic neuron instead, although not in all individuals, indicating that ON1 may possess considerable concentrations of serotonin under unclear circumstances (Hörner et al., 1995). Indeed, there is still no conclusive evidence on the neurotransmitter used by ON1.

The temporal dynamics of ON1 activity might play a crucial role in its function. Wiese and Eilts (1985) suggested that the mutual inhibition of mirror image ON1 is most effective at pulse rates corresponding to that of the species-specific calling song in *G. bimaculatus*. Similarly, Nabatiyan et al. (2003) showed a peak in spike rates at the same temporal pattern. Studies using amplitude modulated sound in different cricket species further support this tuning to the song pattern, suggesting an evolutionary adaptation of the temporal filter properties of ON1 to each species’ own calling song pattern (information coding: Farris et al., 2004; firing rate resonances and computational modeling: Tunstall and Pollack, 2005; Rau et al., 2015). ON1 has also been shown to inhibit the soma-ipsilateral AN1, which is the main relay for conspecific acoustic information to the brain (*Ancistrura*: Molina and Stumpner, 2005; *Acheta*: Stumpner et al., 1995; *Gryllus*: Horseman and Huber, 1994; *Teleogryllus*: Faulkes and Pollack, 2000). Reeve and Webb (2003) hypothesized the inhibition from ON1 might, from a circuit design standpoint, increase the dynamic range of AN1, as well as improve the encoding of the sound onset, therefore decreasing the overall noise in AN1. Nevertheless, it is still not fully clear whether the filter properties of ON1 affect the pattern recognition network in the cricket brain (Schöneich et al., 2015). Interestingly, ON1 with an ascending axon can occasionally occur in various bush cricket and cricket species, and could provide input to the brain alongside AN1 (Atkins and Pollack, 1986; Schul, 1997; Stiedl et al., 1997). However, these data come mostly from nymphs and young adults and are therefore interpreted as an incomplete reduction during development. The overwhelming majority of stained omega neurons in adults do not have an ascending axon and the terminal structures of the observed axons have never been reported.

**FIGURE 3:**
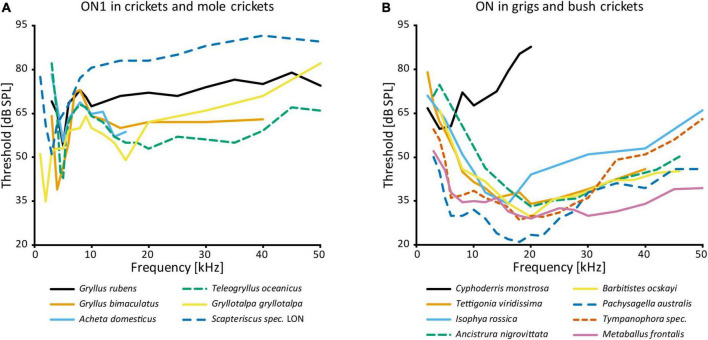
Frequency tuning of ON(1): **(A)** threshold curves of ON1 in crickets and mole crickets. Source of data: *G. rubens*: Farris et al., 2004, *G. bimaculatus*: Watson and Hardt, 1996; *A. domesticus*: Stumpner et al., 1995; *T. oceanicus*: Atkins and Pollack, 1986; *G. gryllotalpa*: Zhantiev and Korsunovskaya, 1990a; *Scapteriscus* spec.: Mason et al., 1998. **(B)** Threshold curves of the omega neuron in grigs and bush crickets. Source of data: *A. nigrovittata* and *B. ocskayi*: Stumpner, 2002; *C. monstrosa*: Mason and Schildberger, 1993; *I. rossica*: Korsunovskaya and Zhantiev, 1992; *Pachysagella australis*, *Metaballus frontalis*, and *Tympanophora* spec.: Römer et al., 1989; *Tettigonia viridissima*: Römer, 1985.

AN1 and AN2, both receiving directional inhibition from ON1, are involved in opposite phonotactic behaviors in response to LF and HF sound respectively. Therefore, multiple studies looked into the frequency-specific processing of ON1. There are two peaks in the frequency tuning of cricket ON1 (with the exception of “high-frequency crickets” Eneopterinae, ter Hofstede et al., 2015): main peak at the calling song frequency and a secondary peak at HF (Popov et al., 1978; Wohlers and Huber, 1978; Atkins and Pollack, 1986; Stumpner et al., 1995; Figure 3A). On top of the fundamental differences in HF vs. LF receptor input into ON1 (Faulkes and Pollack, 2001), information transfer approaches show that high pulse rates are coded much better at HF—as can be found in bat calls—than in LF (Marsat and Pollack, 2004). Such differences may be correlated with the behavior of the animal: *T. oceanicus* is a more active flyer than *G. bimaculatus* and is therefore under stronger predation pressure from bats. A computational model suggested diverse causes that could underlie the frequency-specific responses of ON1, such as cell-intrinsic properties, spike triggered adaptation, interplay between excitation and inhibition, and network-based resonances (Rau et al., 2015). In stark contrast, ON1 in bush crickets does not have conspicuous frequency-dependent differences in input, and its frequency tuning corresponds to that of the whole hearing range except for very low frequencies (e.g., Römer, 1985; Römer et al., 1989; Stumpner, 2002; Figure 3B). This broad tuning is distinct from several other prothoracic interneurons, which are tuned to specific frequencies (e.g., Stumpner, 2002; Triblehorn and Schul, 2009).

Its large and horizontally spread-out branches in crickets make ON1 well suited to study Ca^2+^ dynamics. Ca^2+^ measurements were first used to examine the “cocktail party effect” in *A. domesticus*, demonstrating that forward masking limits the response of ON1 to louder stimuli (Sobel and Tank, 1994). This selective attention phenomenon was reported earlier in *T. oceanicus* (Pollack, 1988). A very similar forward masking/gain control effect was also found in the bush cricket *Tettigonia viridissima* (Römer, 1993; Römer and Krusch, 2000). Both Sobel and Tank (1994) and Römer and Krusch (2000) suggested that Ca^2+^-dependent K^+^ channels inhibit ON1 following activation due to increased Ca^2+^ concentration, which can last for multiple seconds. Finally, a computational analysis corroborated this hypothesis, showing Ca^2+^-dependent spike frequency adaptation and post-synaptic potential depression are sufficient for forward masking (Ponnath and Farris, 2010).

ON1 was the central element in a brilliant experimental setup that enabled electrophysiological recordings in the field (Rheinlaender and Römer, 1986; Römer and Bailey, 1986). This so-called “biological microphone” was used, among others, to record neuronal responses to conspecific calls under natural conditions. Such a setup only works well with rather large neurons, which can be extracellularly recorded in sufficient quality over a longer time even when freely moving the whole setup. Changes in directional responses and neuronal noise depending on the acoustic environment, as well as their behavioral correlates, such as the spacing in the habitat, have been vividly demonstrated with this approach (Römer and Bailey, 1998; Kostarakos and Römer, 2010; Schmidt and Römer, 2011; also see Römer, 2021).

ON1 has also been used to analyze how acoustically active animals solve a common problem, that is strong adaptation of the peripheral nervous system to the animal’s own song. A corollary discharge mechanism leads to primary afferent depolarization and strongly inhibits ON1 activity during singing, thus preserving sensitivity to subsequent external stimuli (Poulet and Hedwig, 2002, 2006). Even when the forewings were removed and the singing was merely fictive, presynaptic inhibition of auditory afferents was in place. Later, the responsible corollary discharge interneuron was also identified and shown to be part of a simple neural network (Poulet and Hedwig, 2007).

Finally, neuronal regeneration following ear lesion was investigated in various cricket neurons, including ON1. When disconnected from auditory receptors, soma-ipsilateral dendrites cross the midline and make new connections on the soma-contralateral neuropile. This process is more extensive in nymphs than in adults, but functionally restores synaptic connections in both cases (Schildberger et al., 1986; Schmitz, 1989). Such plasticity seems to be restricted to first-order interneurons (Lakes, 1990; Lakes et al., 1990). Therefore, the changes can be seen as evidence that ON1 receives monosynaptic input from the auditory receptors. Interestingly, some plasticity can also occur after soma-contralateral lesions. Since post-lesion changes are only seen in branches with direct input from afferents, ON1 has direct input from both ears, supporting electron microscopy data showing synapses with profiles matching those of sensory neurons on both sides of TG1 (Watson and Hardt, 1996). Weak excitation from the “inhibited” side following acute lesions has been reported in other directional orthopteran interneurons as well (Lakes et al., 1990).

A post-lesion regenerated ON1 is morphologically strikingly similar to ON2, which has another crossing segment within the auditory neuropile. ON2 occurs in multiple cricket species (*A. domesticus*: Atkins et al., 1984; *G. bimaculatus*: Schmitz, 1989; *G. campestris*: Wohlers and Huber, 1982; *Gryllus rubens*: Farris et al., 2004; *T. oceanicus*: Lewis, 1992). As for ON1, occasional thin ascending axons occur in ON2, but no terminals were stained (*A. domesticus*: Stiedl et al., 1997; *G. bimaculatus*: Schmitz, 1989). One staining in the grig *C. monstrosa* includes two omega neurons within the same hemiganglion, where one cell has a thin neurite crossing the midline, indicating this is ON2 (Mason and Schildberger, 1993). However, ON2 does not occur in any bush cricket species, even though Tettigoniidae (bush crickets) and Prophalangopsidae (which grigs belong to) share around 100 million years of common evolution after splitting off from the gryllid line (Song et al., 2015, 2020). A parsimonious explanation then would be that bush crickets lost ON2 secondarily. However, since the common ancestor of grigs and bush crickets had hearing, this loss must have happened in an active, established auditory processing network.

**FIGURE 4:**
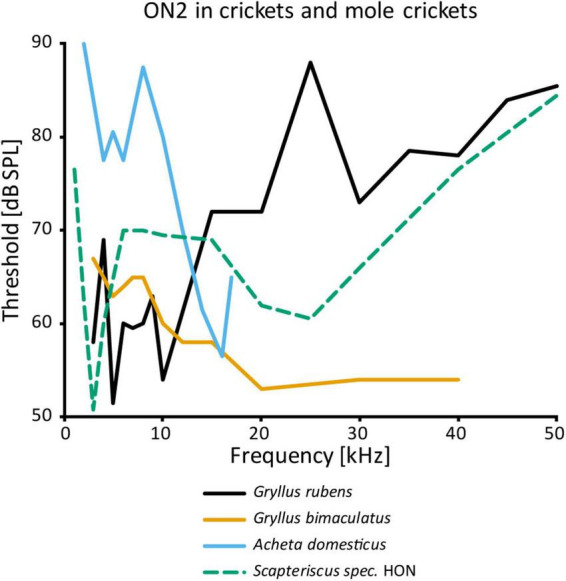
Frequency tuning of ON2 in female crickets and “high-frequency-tuned omega neuron” in a mole cricket. Source of data: *G. rubens*: Farris et al., 2004, *G. bimaculatus*: Watson and Hardt, 1996; *A. domesticus*: Stiedl et al., 1997; *Scapteriscus* spec.: Mason et al., 1998.

While the physiology of ON1 is consistent across different taxa, that of ON2 varies considerably, even within the same genus (Figure 4). The auditory response was shown to have lower thresholds for LF than HF in *G. campestris* and *G. rubens* (Wohlers and Huber, 1982; Farris et al., 2004, respectively). Yet, in regeneration experiments in *G. bimaculatus* it had similar thresholds for both frequency ranges (Schmitz, 1989), while Watson and Hardt (1996) reported distinctly higher sensitivity to HF in the same species. The latter is congruent with data from other cricket species (*A. domesticus*: Stiedl et al., 1997; *T. oceanicus*: Lewis, 1992). However, all studies agree that ON2 receives excitatory input from both ears and has little directionality. With an elegant experimental approach using selective cold-inactivation of ears, Zhang and Hedwig (2019) could directly demonstrate bilateral excitatory input to ON2 in *G. bimaculatus*, consistent with earlier electron microscopy data (Watson and Hardt, 1996). Several studies reported that ON2 does not copy the temporal pattern of the conspecific song very well (e.g., Wohlers and Huber, 1982). Mason et al. (1998) identified two omega neurons in the mole cricket genus *Scapteriscus*. Although morphologically indistinguishable, these neurons differ in their frequency responses: one is tuned to LF, the other is additionally sensitive to HF. Therefore, the authors name them low and high-frequency-tuned omega neurons, and compare them to ON1 and ON2, respectively. No such differences were reported for any species in the only other extant mole cricket subfamily Gryllotalpinae.

Since ON1 provides inhibitory input in the prothoracic auditory network, ON2 could play a similar role. However, electron microscopy data show clear differences in the synaptic vesicles between ON1 and ON2, suggesting different neurotransmitters (Watson and Hardt, 1996). Like ON1, ON2 is not GABAergic. Inhibition by HF sound has been shown in ascending neurons in *G. campestris* (Boyd et al., 1984) and *A. domesticus* (Stumpner et al., 1995), with ON2 as a possible source. In the latter species, the inhibition remained after eliminating all soma-contralateral input, pointing to an ipsilateral source.

## Dorsal unpaired median neurons

**FIGURE 5:**
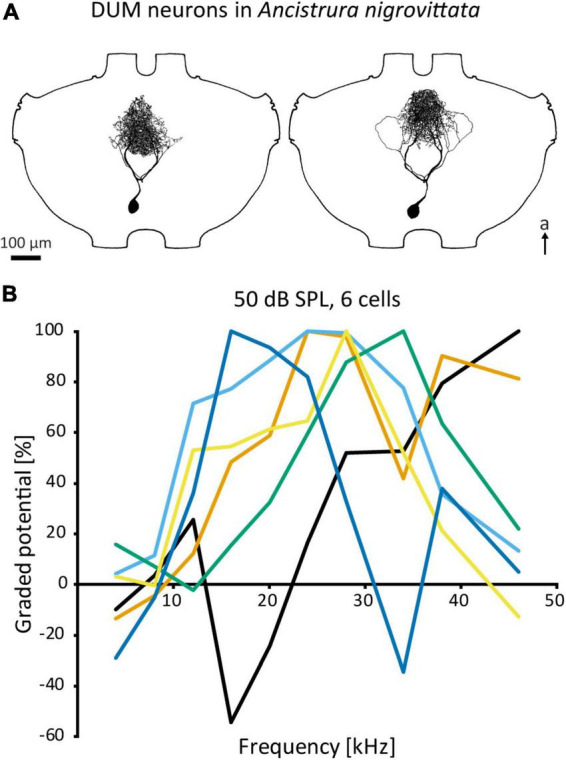
DUM neurons in *A. nigrovittata*. **(A)** Two DUM neurons in males, the left one is morphologically classified as “narrow” and high frequency tuned, the right one is classified as “loops” and mid frequency tuned. **(B)** Iso-intensity responses of 6 out of 11 DUM-neurons recorded in one female with the relative graded responses (sum of excitation and leading inhibition normalized to the maximum response in each curve). Each cell has a different frequency tuning. a, anterior.

Unpaired median neurons constitute a class defined by the medial position of their cell bodies, forming a cluster at the posterior end of thoracic and abdominal ganglia (e.g., Hoyle, 1978; Lange and Orchard, 1984; Janiszewski and Otto, 1988; see Figure 5A). Unpaired median neurons occur across the dorsoventral axis and the distinction between the dorsal and ventral cells is usually artificial (Bräunig and Pflüger, 2001). Therefore, we will not differentiate unpaired neurons on this basis and will use the term “DUM neuron” for all such cells.

Although all DUM neurons within the same ganglion originate from the same neuroblast (Goodman and Spitzer, 1979), they are heterogeneous both in terms of morphology (e.g., cell body size, projection area) and function (e.g., neurotransmitter, sensory modality, role in behavior). DUM neurons occur in a variety of taxa, but were only investigated in detail in cockroaches (Tanaka and Washio, 1988; Washio, 2002) and orthopterans, especially grasshoppers. Within orthopterans, there are two distinct DUM neuron populations. The first group consists of neurons that have large cell bodies, project into the peripheral nerves or to other segments, are octopaminergic, and are commonly associated with neuromodulatory or motor control functions (Hoyle, 1975, 1978; Gras et al., 1990; Thompson and Siegler, 1991). The second and more numerous group has neurons that have smaller cell bodies, project mostly within the ganglion and rarely into connectives, and are immunoreactive for antibodies against GABA (Thompson and Siegler, 1993; Stumpner et al., 2020). Though DUM neurons have been extensively investigated regarding their neuromodulatory function or electrical properties (e.g., Grolleau and Lapied, 2000; Bräunig and Pflüger, 2001), there is limited data on their role in sensory processing. Diverse and evolutionarily far groups within Orthoptera have DUM neurons responsive to sound and/or vibration (grasshoppers: Marquart, 1985; Stumpner and Ronacher, 1991; Thompson and Siegler, 1991; crickets: Gras et al., 1990; cave crickets: Stritih and Stumpner, 2009; bush crickets: Lefebvre et al., 2018). Yet, the only detailed studies on auditory DUM neurons have been in the prothoracic ganglion of *A. nigrovittata* (Lefebvre et al., 2018; Stumpner et al., 2019, 2020).

Auditory DUM neurons in *A. nigrovittata* constitute a heterogenous group with multiple morphological types, which correspond to their physiological response properties only to a limited extent (Lefebvre et al., 2018; also Figure 5A). Some types have extensive arborizations within the auditory neuropile and are sensitive to airborne sound, while other DUM types project also or exclusively to ganglion regions outside the auditory neuropile and can be sensitive to vibration. Auditory DUM neurons differ significantly in their frequency tuning (Figure 5B). Different cells have different best frequencies and this tuning is sharpened by the extensive frequency-dependent inhibition (Lefebvre et al., 2018). The population includes 15 or more cells, covers a wide frequency range, and is thought to constitute a filter bank. This proposed function extends to temporal processing as well (Stumpner et al., 2019). Their diverse filtering properties and inhibitory output make DUM neurons the main candidates for inhibitory effects—especially frequency dependent inhibition—in auditory interneurons within the prothoracic network. Therefore, they could represent a major part of the early sensory processing.

## Segmental neurons

The term “segmental neuron” (SN) denotes some local interneurons that are branching mostly within one hemiganglion of the central nervous system. The first auditory SN were described in *Locusta migratoria* (SN1 and SN2, Römer and Marquart, 1984). Like DUM neurons, SN are only defined by their morphological features. They vary considerably in their morphology, such as projection areas, as well as physiological properties, and do not constitute a functional class.

In total, four auditory SN have been reported in three ensiferan species: two cells in *A. domesticus* (local neuron (LN) 1 and 2, Stiedl et al., 1997; Figure 1A), and one each in the bush crickets *I. rossica* (SN1, Zhantiev and Korsunovskaya, 1990b) and *A. nigrovittata* (SN2, Stumpner, 1995) (Figure 1B). SN2 also occurs in several *Barbitistes* species (A. Stumpner, unpublished data). In contrast to the SN described in the grasshopper *L. migratoria*, which spread over both sides of the ganglion, segmental neurons in ensiferans have the majority of their arborizations within a single hemiganglion. LN1 and LN2 in *A. domesticus* are both non-spiking and tuned to low frequencies around the carrier frequency of the species calling song (∼5 kHz) (Stiedl et al., 1997). LN1 is inhibited by high-frequency sound, whereas LN2 is inhibited by vibration, but activated by wind. SN1 in *I. rossica* responds to 12–16 kHz sounds very sensitively (<30 dB SPL) with tonic spike trains (Zhantiev and Korsunovskaya, 1990b; Korsunovskaya and Zhantiev, 1992). Intriguingly, the projection area of SN1 lies completely outside the auditory neuropile, since there is no overlap with the branches of ON1 from the same species (Zhantiev and Korsunovskaya, 1990b; Korsunovskaya and Zhantiev, 1992). This suggests an auditory input coming exclusively from other interneurons, which is unusual for local neurons. Information on SN2 up to now has only been cursorily reported (Stumpner, 1995; Stumpner and Nowotny, 2014).

**FIGURE 6:**
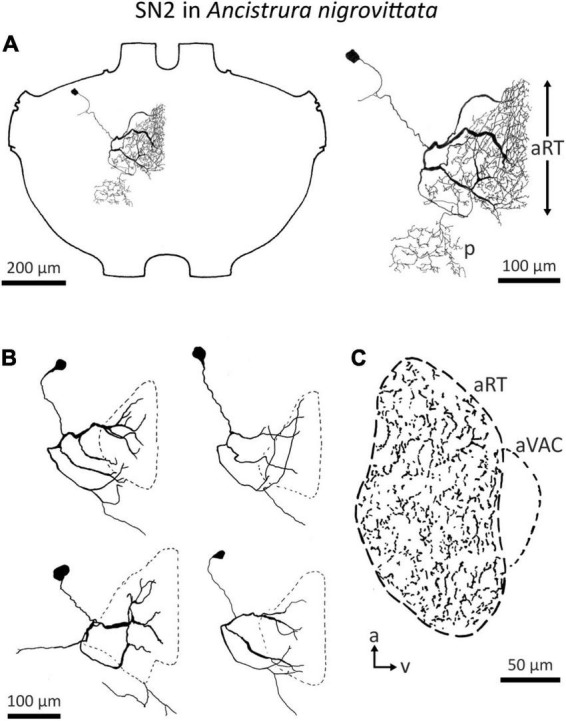
Morphology of SN2 in *A. nigrovittata*. **(A)** Wholemount and detailed view of SN2 in a female. The extent of the anterior ring tract (aRT) is indicated by the arrows; the secondary branching area marked with p lies posterior and more laterally to the aRT. **(B)** Four examples of the main branches in two females (upper) and two males (lower). The extensions of the dendritic trees are indicated by a dashed line. **(C)** Parasagittal section (16 μm) close to the midline showing the arborizations of SN2 in a female. The dashed line delimits the auditory neuropile, corresponding to aRT, the smaller dashed line on the ventral side indicates the anterior ventral association center (aVAC; e.g., Lakes and Schikorski, 1990).

Detailed morphological and physiological data on SN2 exist only in *A. nigrovittata*. SN2 is a local auditory interneuron with a lateral cell body and extensive arborization within the auditory neuropile, as well as a secondary, more posterior projection area (Figure 6A). This area is situated posterior to the ventral median tract (VMC) and lies in the approximate position of the supra median commissure (SMC, Wohlers and Huber, 1985; Lakes and Schikorski, 1990). SN2 can differ significantly in the details of their posterior branches (Figure 6B). Yet, they all have the auditory neuropile as their primary projection area. This projection completely covers the neuropile (Figure 6C).

**FIGURE 7:**
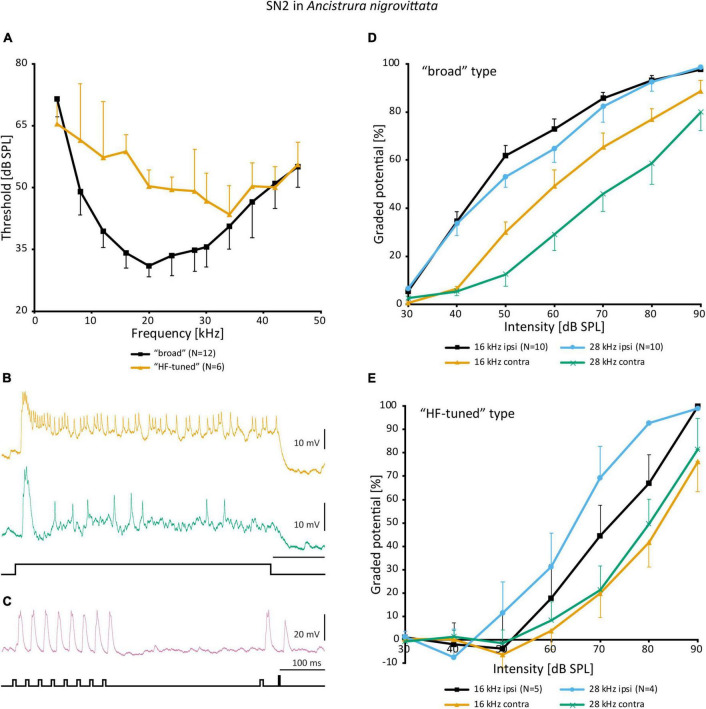
Physiology of SN2 in *A. nigrovittata*. **(A)** Frequency tuning (mean ± SD) of “broad” (black; 8 females, 4 males) and “HF-tuned” SN2 (orange; 3 f, 3 m). **(B)** Response patterns of two SN2 in males to a 500 ms white noise stimulus of 70 dB SPL. Upper trace from a “broad”, lower from an “HF-tuned” neuron. **(C)** Response of a “broad” SN2 to an artificial duet between a male (smaller pulses, 16 kHz) and a female (larger single pulse, 28 kHz) at 60 dB SPL. **(D,E)** Intensity response curves for soma-ipsilateral (ipsi) and soma-contralateral (contra) 100 ms stimuli at 16 and 28 kHz (mean ± SEM). **(D)** “Broad” neurons (6 f, 4 m). **(E)** “HF-tuned” neurons (16 kHz: 3f, 2m; 28 kHZ: 3f, 1 m).

**FIGURE 8:**
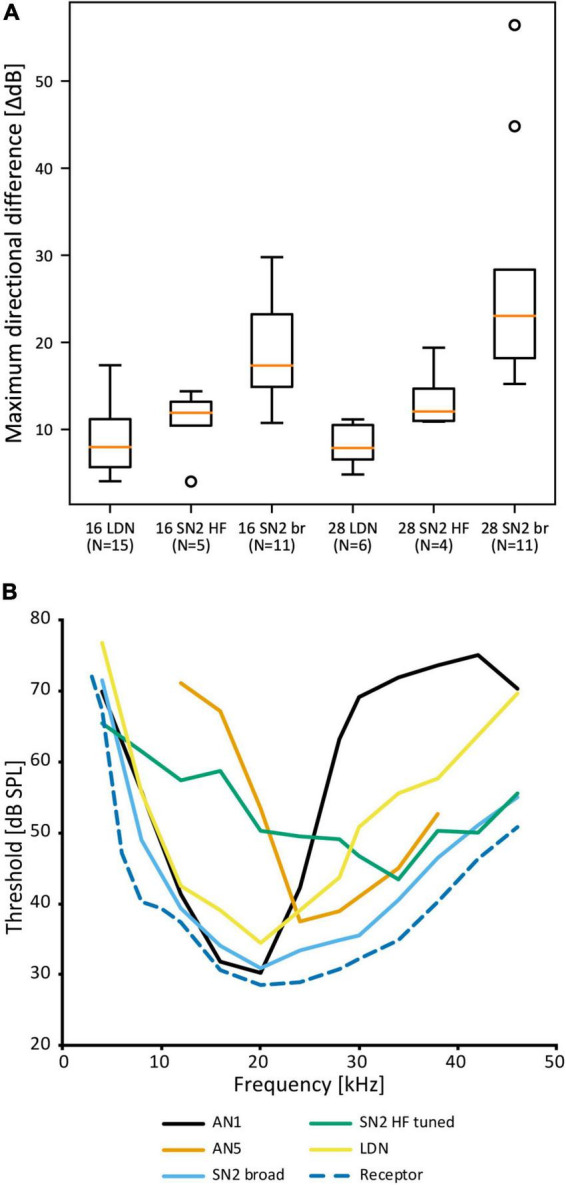
Comparison of SN2 and LDN to other neurons in *A. nigrovittata*. **(A)** Box plot (median in orange, lower and upper quartile; whiskers represent the minimum/maximum value within 1.5 times the interquartile range; outliers shown as circles) of the maximum difference in dB between the responses to ipsilateral and contralateral stimulation measured within the dynamic range of intensity scans as shown in Figures 7D,E. 16 and 28 represent the tested carrier frequencies in kHz; SN2 are divided as “broad” (br) and “HF-tuned” (HF). **(B)** Frequency thresholds of LDN and SN2 in comparison to the overall hearing threshold (minimal values from auditory receptor neurons; see Ostrowski and Stumpner, 2010) and that of the spikes of the ascending neurons tuned best to the male song (AN1, mean of 21–23 (30 kHz: 10) males and females) or tuned best to the female song (AN5-AG7, mean of 4–8 males; see Stumpner and Molina, 2006).

Despite the notable variation, there are no distinct morphological subtypes of SN2. Yet, physiological data can be categorized in two groups. “Broad” SN2 have a broadband frequency tuning with the lowest thresholds around 20 kHz. “HF-tuned” SN2 share the > 35 kHz section of their tuning with the “broad” type, but are on average much less sensitive to low-frequency sound and show larger interindividual variation than “broad” SN2 (Figure 7A). Basic response patterns of SN2 can vary greatly. Though all SN2 share an underlying phasic-tonic motif, the ratio between the phasic and tonic portions changes significantly between cells (Figure 7B). Even the presence or absence of spikes can differ between recordings (all recordings were done in or close to the auditory neuropile). Nevertheless, SN2 can reliably represent the species-specific calling song, although the relative response strength to male and female calls varies (Figure 7C). “Broad” SN2 have similar intensity response curves for frequencies between 16 and 28 kHz and a wide dynamic range spanning the entirety of the tested stimulus space (30–90 dB SPL) (Figure 7D). In contrast, “HF-tuned” SN2 show higher activity for 28 kHz than 16 kHz (Figure 7E). The dynamic range of the “HF-tuned” SN2 could not be revealed, as– likely due to the high thresholds—the maximum stimulus intensity did not saturate the neuron. “Broad” SN2 are much more directional than the “HF-tuned” at 16 and 28 kHz, with maximum response difference between ipsi- and contralateral side reaching >40 dB (median: 16 kHz: 17.3 vs. 11.9 dB; 28 kHz: 23.0 vs. 12.1 dB, respectively; Figure 8A). Data present a complicated picture for SN2: there seem to be two physiological subtypes without consistent morphological delimitation. In one individual, two SN2 with adjacent cell bodies on the same hemiganglion and similar frequency tuning (both “broad”) were stained, suggesting the existence of more than one cell on each side. The broad variety has been recorded twice as often as the HF-tuned within the dataset (23 cells in total, 18 with complete physiology, compare Figure 7A). This could be interpreted as there being three SN2 on each side of the prothoracic ganglion: two broad and one HF-tuned SN2. So far, only two SN2 have been stained in the same hemiganglion.

## “Local descending neuron”

**FIGURE 9:**
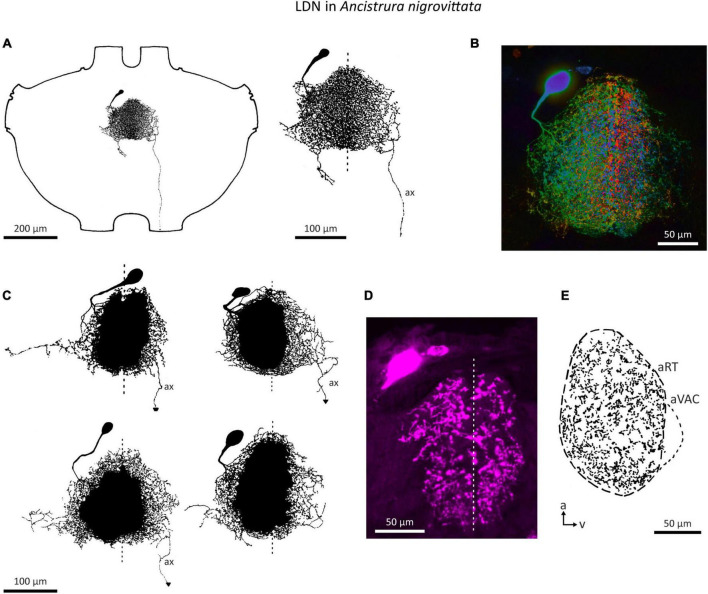
Morphology of the local neuron LDN in *A. nigrovittata*. **(A)** Wholemount and detailed view of LDN in a male. **(B)** Color coded (warm = dorsal, cold = ventral) maximum projection of a confocal stack of LDN filled with neurobiotin and developed with streptavidin-Cy3 in a female. **(C)** Four examples of the main branches in two males (upper) and two females (lower). A dashed line indicates the ganglion midline. **(D,E)** Sections (10 μm) of LDN in a female **(D)** and a male **(E)**. **(D)** Confocal image of a transversal section showing the similarity of branches in both hemiganglia. **(E)** Drawing of a parasagittal section showing the arborizations in the auditory neuropile. a, anterior; aRT, anterior ring tract; aVAC, anterior ventral associations center; ax, axon-like branch; v, ventral.

An auditory interneuron has been characterized in *A. nigrovittata* and coined “local descending neuron” (LDN). Though a contradictory name, we believe it represents the morphological properties of this cell type rather accurately. LDN is similar to a descending neuron in *A. nigrovittata* (“DN4,” Stumpner and Nowotny, 2014) and two descending neurons from *Decticus albifrons* (Sickmann, 1997). One LDN occurs on each side of the prothoracic ganglion, with the cell body in an anteromedian cluster of somata, adjacent to those of other descending neurons (Stumpner and Nowotny, 2014; A. Cillov, unpublished data; also Figure 9A). LDN has dense and extensive arbors in the auditory neuropile (Figures 9B–E). Unlike other DN, the primary neurite of LDN splits into fine branches upon entering the auditory neuropile without a crossing segment or axon running through the arborizations. In 12 out of 21 stains, a fine projection originates from the contralateral branches and terminates before reaching the connective. Only in one case the projection reached the connective, but it ended before reaching the mesothoracic ganglion. We interpret this projection as a rudimentary axon. This could be a case of deterioration in the course of development, though two subadult animals had similarly thin and prematurely terminating axons. LDN has some interindividual morphological variety in the projection of its lateral branches (Figure 9C). The branching pattern is always on both sides of the ganglion with no clear difference in the size or shape of the dendrites (Figure 9D). Like SN2, LDN projects to the entire auditory neuropile and the dense branches are mostly restricted to the neuropile (Figure 9E).

**FIGURE 10:**
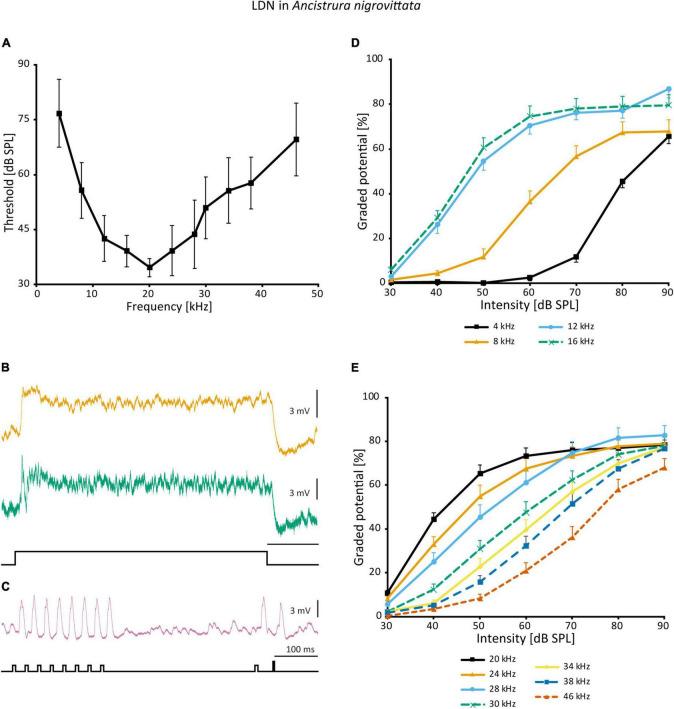
Auditory responses of LDN in *A. nigrovittata*. **(A)** Frequency tuning (mean ± SD) of LDN from 9 to 10 females and 7 to 8 males. **(B)** Response patterns of two LDN in a female (upper) and a male (lower) to a 500 ms white noise stimulus of 70 dB SPL. **(C)** Response of an LDN in a female to an artificial duet between a male and a female at 60 dB SPL. **(D,E)** Intensity response curves for the indicated frequencies with 50 ms stimuli (mean ± SEM; 10 females and 8 males, except for 8 kHz (9f, 7 m) and 38 kHz (10 f, 7 m). Response curves up to 16 kHz **(D)** have a steeper rise and a narrower dynamic range than those in the ultrasound **(E)**.

In the frequency domain, LDN is broadly tuned, though overall less sensitive than SN2 and has its peak around 20 kHz (Figure 10A). The responses of different LDN are much more consistent than those of SN2. LDN is non-spiking and responds to vibration little if at all, and with acoustic stimuli, it is a phasic-tonic neuron (Figure 10B). In most cases, a phasic fall of the cell potential occurs shortly after the onset of excitation, the extent of which varies between cells (Figure 10B). LDN faithfully copies the species’ duet between the male and female (Figure 10C).

A non-linearity occurs in the intensity response curves of different frequencies. Frequencies <20 kHz (Figure 10D) have a steeper slope and a narrowed dynamic range compared to frequencies >20 kHz (Figure 10E). Unfortunately, it is not possible to directly calculate and compare dynamic ranges, as responses on both ends of the spectrum have not reached saturation within the tested intensity range. This could imply that different inputs into LDN are weighted differently depending on the frequency or that there is additional polysynaptic input at HF, which, however, is not indicated by the latencies. Such a finding would also suggest that a wider dynamic range is of particular importance to the unidentified postsynaptic targets.

Auditory interneurons in *A. nigrovittata* are clearly directional (e.g., ON1, AN1, TN1; Stumpner and Molina, 2006). LDN, however, has remarkably small response differences between stimuli from opposite directions. The maximum dB difference in response to left and right stimulation for LDN is ∼8 dB for 16 and 28 kHz, the frequencies of male and female song in *A. nigrovittata*. This is noticeably lower than for both SN2 subtypes (Figure 8A).

Leg cut experiments with “broad” SN2 have diverse results: only input from the ipsilateral ear, inhibition from the contralateral ear, or clear excitation from both ears. The dB difference in LDN is also lower than that in sensory neurons (13–17 dB; Lefebvre et al., 2018), and similar to that of DUM neurons, which are excited by both ears (Lefebvre et al., 2018).

## Discussion

Local auditory interneurons in the prothoracic ganglion represent a major part of the first level of information processing in ensiferan insects and still harbor unexplored complexity. Data across the breadth of ensiferan taxa show that bush crickets have by far the highest diversity of local auditory neurons. This could be confounded by the focus on a subset of bush crickets, namely Phaneropterinae, as other bush cricket subfamilies (e.g., Tettigoniinae) do not seem to exhibit the same variety of local auditory cells.

ON(1) is the only local neuron found across all investigated ensiferan taxa and thus is a good starting point for comparisons between groups. ON(1) seems to be both morphologically and physiologically, conserved throughout Ensifera, as all records share certain hallmarks. ON(1) is always highly directional due to mutual contralateral inhibition. In addition, it is involved in sound localization by inhibiting the ascending neuron(s) that receive their main excitatory input from the soma-contralateral ear. Other proposed functions are gain control and coding sound onset more precisely, though none have been shown directly. Temporal tuning of ON(1) demonstrates evolutionary adaptation to each species’ own calling song (Farris et al., 2004; Tunstall and Pollack, 2005; Rau et al., 2015), though it is also involved in general sound source localization, including that of predators (Selverston et al., 1985; Schildberger and Hörner, 1988).

At least two other local neurons are likely to be inhibitory. ON2 could be involved in inhibition more pronounced at high frequencies. In crickets, this could help with the separation of conspecific (LF) vs. predator (HF) auditory channels. In contrast, GABAergic DUM neurons in (certain) bush crickets provide a much more granular filter bank for frequency-specific inhibition. This fine separation could help code the difference between the auditory channels of the male and female signals, which can be at different frequencies as part of duets, as in *A. nigrovittata*. Fine separation might also allow sexual selection by fitness or size correlated song parameters, although such effects are hard to demonstrate in Orthoptera (e.g., Shaw and Herlihy, 2000; Verburgt and Ferguson, 2010). A broad filter bank for frequency specific inhibition could facilitate rapid speciation through changes in the calling song frequency. Such cladogenesis events are known to have occurred in the bush cricket evolutionary line, though presumably due to geographical separation (e.g., Heller et al., 2011). Immunohistochemical data show that crickets also have a GABAergic DUM cluster at the same position as bush crickets, but these likely have a non-auditory, possibly vibratory function (Cillov, 2020).

Data on segmental neurons is exceedingly patchy. LN1/LN2 and SN1 are only known from single species (*A. domesticus* and *I. rossica*, respectively); SN2 from two closely related genera (*Ancistrura* and *Barbitistes*). SN1 is intriguing due to its morphology and is unlike any other known local auditory neuron in Ensifera. Both LN1 and LN2 are tuned to low frequencies and are sensitive enough to be involved in intraspecific communication. If LN exist in other cricket groups and are excitatory, they could be the source of the LF polysynaptic input to ON1 (Faulkes and Pollack, 2001), for which there are no other candidates among local auditory neurons. If they are inhibitory, they could be the LF counterparts to ON2 and provide LF inhibition to ON2 and AN2. Any inhibition in the frequency or temporal domain in bush crickets is likely to be fulfilled by DUM neurons, which cover a wide frequency range when taken as a whole.

Local descending neuron is known from a phaneropterid species (*A. nigrovittata*), though similar neurons of prothoracic origin with projections in posterior ganglia are known from several bush cricket species (Sickmann, 1997; Kostarakos and Römer, 2015). SN2 and LDN are both unlikely to contribute to song recognition, as neither provides any conspicuous filtering in the frequency or temporal domain. Only two SN2 members had signs of inhibition in high frequencies. As ON(1) already is a source of broadband inhibition, one might except SN2 and LDN to be excitatory, but there are no conclusive results from immunohistochemical experiments.

LDN and SN2—especially the “broad” subtype—could function as reference neurons. They would represent the whole auditory spectrum without any obvious filtering and represent the presence of sound. “Broad” SN2 have a frequency tuning like the most sensitive auditory receptor cells, just with few dB higher thresholds (Figure 8B). LDN is even less sensitive with more pronounced interindividual differences in the ultrasound. “Broad” SN2’s tuning encompasses those of the most specific intersegmental neurons for male and female song in *A. nigrovittata* (AN1 and AN5-AG7, respectively; Figure 8B and Molina and Stumpner, 2005). LDN and SN2 HF complement each other by being more responsive to lower and higher frequencies, respectively. Neurons with similar broad tuning as SN2 are found among ascending neurons in several Orthoptera, though their roles in the greater network are unknown (AN3 in bush crickets: Stumpner and Molina, 2006; AN6 in Caelifera: Römer and Marquart, 1984; Stumpner and Ronacher, 1991). A role of such neurons could be in multimodal integration between acoustics and wind or vibration. A problem with the reference neuron hypothesis is the rarity of such neurons. One example is in the primate auditory cortex (Brasselet et al., 2012), but in this framework, reference neurons are marked for their low and precise latencies, which is not indicated for SN2 and LDN.

With a soma diameter of 30 μm or more, LDN is reminiscent of neuromodulatory cells, such as octopaminergic DUM neurons, which have big cell bodies. Combined with its dense arborization throughout the auditory neuropile, LDN could be a candidate for modulating auditory processing. However, previous work did not find any hints for anterior cell bodies with biogenic amines like serotonin or octopamine in bush crickets (A. Stumpner, unpublished data) nor in crickets (Hörner et al., 1995). Octopamine, however, like histamine, influences the responses of ON1 in crickets (Skiebe et al., 1990; Lühr et al., 1994). The low interindividual variability of most prothoracic auditory neurons does not support a strong neuromodulatory influence.

To conclude, though the insect central nervous system is simpler than that of vertebrates, we are unable to even reveal the early “subcortical” networks in a taxon that has been continuously studied for over 50 years. Though in Ensifera, the data also suffer from fragmentation over several groups. Yet, even in *G. bimaculatus*, which is the most intensively studied species, neither the neurotransmitters of auditory neurons nor their connectivity to each other is known, except for a handful of cases. It is even likely there to be undiscovered auditory neurons relevant for behavior. We severely need data on components of the local auditory processing network other than ON. With the tools available right now, these are mostly limited to electrophysiological data. Yet, they may enable us to understand the exact neuronal mechanisms underlying auditory perception, as well as to elucidate the evolutionary pressures and processes shaping the nervous system and speciation in this group with sophisticated acoustic communication. Though one thing is certain: even the fragments we have hint at a system much more complex than we are aware of at the moment.

## Materials and methods

Figures 1–5 present in parts so far unpublished data, Figures 6–10 new data. The methods are described in short, but are in detail in Lefebvre et al. (2018).

Animals: *Ancistrura nigrovittata* (Brunner von Wattenwyl, 1878) were caught in Northern Greece and reared in the laboratory for up to 9 generations. *Barbitistes serricauda* (Fabricius, 1798) were F1-generations originating from Lower Saxony, Germany. *Barbitistes ocskayi* (Brunner von Wattenwyl, 1878) were F1-generations from southwestern Slovenia. *Gryllus bimaculatus* (De Geer, 1773) came from a laboratory culture that existed for many years in the Zoological Institute of the University of Göttingen. *Pholidoptera griseoaptera* (De Geer, 1773) were caught in Göttingen, Germany.

Neuron morphologies were revealed by intracellular stainings with Lucifer Yellow CH, Alexa 555 Hydrazide or neurobiotin, which was coupled to streptavidin-Cy3. Neurons were either drawn from an epifluorescent microscope with a drawing tube or from confocal images (Leica SP8 AOBS, maximum projections of z-stacks and single images). All neuronal morphologies were transferred into standard ganglia for crickets or bush crickets for better comparability. For comparative figures unpublished stainings are shown whenever available. In the remaining cases, neurons were redrawn from publications (photos, drawings) for a homogeneous design. For histological analysis, ganglia were embedded in Agar 100 and sectioned (10–16 μm). Neuronal projections were drawn from microscope and confocal images.

Physiological data for DUM neurons, SN2, and LDN were recorded with a standard intracellular bridge-amplifier (NPI, Germany), stored on DAT-tape (SONY, Japan) or directly digitized using a commercial AD-converter and the software Spike2 (CED, UK). Data were analyzed with custom-written scripts in Spike 2. Graded potentials are given as area (positive or negative; mV * ms) between resting potential and actual membrane potential (spikes clipped) during the response to a stimulus and normalized to the maximum response. Stimuli were presented using a custom-made setup. Stimulus envelopes (1.5–2 ms rise and fall times) were filled with sine waves or white noise (ca. 2–50 kHz) and repeated 5 times (except for the long white noise stimuli from Figures 7A, 10A). Data points in the frequency threshold curves show individual means of several animals and standard deviation (SD), individual values were calculated once from frequency-intensity scans. In all other cases, the means and standard errors (SEM) are shown, each data point is the mean of the averaged measurements from different individuals.

## Data availability statement

The raw data supporting the conclusions of this article will be made available by the authors, without undue reservation.

## Author contributions

AS made the neuron drawings, replotted data from previous studies, gathered the literature, generated the SN2 data, and provided the cookies. AC made the figures and generated the LDN data. Both authors wrote the manuscript.
